# Removal and Recovery of AgNPs from Water by Sustainable Magnetic Nanoflocculants

**DOI:** 10.3390/polym17050650

**Published:** 2025-02-28

**Authors:** Mariana Ramirez, Eya Ben Khalifa, Giuliana Magnacca, Mario Sergio Moreno, María E. Parolo, Luciano Carlos

**Affiliations:** 1 Instituto de Investigación y Desarrollo en Ingeniería de Procesos, Biotecnología y Energías Alternativas, PROBIEN (CONICET-UNCo), Universidad Nacional Del Comahue, Buenos Aires 1400, Argentina; mariana.ramirez@probien.gob.ar; 2 Department of Chemistry and NIS Research Centre, University of Torino, Via P. Giuria 7, 10125 Torino, Italy; eya.benkhalifa@unito.it (E.B.K.); giuliana.magnacca@unito.it (G.M.); 3 Instituto de Nanociencia y Nanotecnología, INN (CNEA-CONICET), Centro Atómico Bariloche, Av. Bustillo, San Carlos de Bariloche 8400, Argentina; smoreno@cab.cnea.gov.ar; 4 Centro de Investigaciones en Toxicología Ambiental y Agrobiotecnología del Comahue, CITAAC (CONICET-UNCo), Facultad de Ingeniería, Universidad Nacional Del Comahue, Buenos Aires 1400, Argentina; maria.parolo@fain.uncoma.edu.ar

**Keywords:** magnetic nanomaterials, biopolymers, flocculants, water treatment, silver nanoparticles

## Abstract

The presence of silver nanoparticles (AgNPs) in water bodies has emerged as a new environmental concern and the efficient separation of these nanoparticles remains a critical challenge. Here, we developed novel magnetic nanoflocculants for the recovery of AgNPs from water. Alternating layers of biopolymers, in particular, chitosan, alginate, and polymeric bio-based soluble substances (BBS) derived from urban waste, were coated on magnetic nanoparticles via the layer-by-layer technique to prepare reusable magnetic nanoflocculants (MNFs). The MNFs obtained were characterized with diverse physicochemical techniques. Surface response methodology, based on the Doehlert matrix, has shown to be a useful tool to determine the effect of pH (in the range 5–9), concentration of AgNPs (7–20 mg L^−1^), and MNFs (50–1000 mg L^−1^) on the performance of AgNPs removal. The model predicts a high AgNPs removal percentage at low pH values and high MNF concentration. In particular, for the most efficient MNFs, 90% of AgNPs removal was obtained at pH 5 and 600 mg L^−1^ MNF concentration. Additionally, the effects of AgNPs size, ionic strength, the presence of humic acids, and two types of surfactants (LAS anionic and TWEEN 20 nonionic) on the AgNPs removal were evaluated. Finally, recovery and reuse experiments showed that MNF made of Chitosan-BBS can be reused in ten cycles, losing only 30% of the initial removal capacity. Therefore, magnetic flocculation could represent a sustainable alternative for AgNPs separation with potential applications in water treatment and remediation of nanoparticle contamination.

## 1. Introduction

In recent years, the use of silver nanoparticles (AgNPs) has increased significantly due to their wide range of applications, particularly for their antimicrobial, electrical, and optical properties [1]. According to the Nanotechnology Products Database, AgNPs have been incorporated into 365 different product types, including wound dressing, ink, air purifiers, water purifiers, food packaging materials, food storage containers, dietary supplements, laundry detergents, and lotions and soaps, among others [2]. Consequently, AgNPs are among the most commercialized engineered nanomaterials [3]. Numerous studies report on the release of AgNPs from consumer products into the environment, emphasizing their potential risks [4,5]. Evidence has shown that AgNPs pose significant threats to a variety of organisms, including bacteria, algae, fungi, invertebrates, plants, and fish [6,7]. Notably, AgNPs can leach and accumulate in natural waters, causing high potential risk of Ag contamination [8]. For these reasons, the development of effective remediation processes to remove and recover AgNPs from water is crucial to preserve aquatics ecosystems.

Despite advances in separation technologies, the effective removal of nanoparticles from aquatic environment remains a significant challenge. Colloidal nanoparticles are often stably suspended in solution, resisting gravitational sedimentation for extended periods. Disrupting these stable suspensions typically requires intensive and costly processes such as membrane filtration [9]. On the other hand, among the solid–liquid separation processes, coagulation–flocculation is one of the most used due to its cost-effectiveness and easy operation. This water treatment technology improves the settling performance and increases the sedimentation rates of the suspended solids in water by introducing coagulants that destabilize the charged particles by neutralizing their surface charges, leading to the formation of larger and denser flocs. Inorganic coagulants such as aluminum- and iron-based salts, and organic coagulants, such as polyacrylamide, are commonly used in a traditional coagulation–flocculation process [10]. However, the use of organic biopolymers obtained from natural sources or waste is gaining considerable attention as a new class of coagulants due to their wide availability, low price, non-toxicity, and biodegradability [11]. For instance, chitosan, a cationic biopolymer derived from chitin, the most abundant natural amino polysaccharide in nature, is a very promising coagulant that has been used in the treatment of various wastewaters containing different pollutants [12]. On the other hand, alginates, natural polysaccharides extracted mainly from brown algae, were also used as potential anionic biopolymeric coagulants in a coagulation–flocculation process [13]. Aerobic biodegradation of the wet organic fraction of municipal waste has been shown to produce polymeric bio-based substances (BBS), which exhibit chemical similarities to humic substances [14]. BBS have been identified as mixtures of molecules with varying molecular weights, ranging from 67 to 463 kg mol^−1^ [15]. These molecules consist of aliphatic carbon chains and aromatic rings substituted with various functional groups, including -COOH, -CON, -C=O, -OH, and -O-alkyl, among others. These BBS possess a net negative charge making them suitable for use as green auxiliary reagents in various functionalized nanoparticles synthesis processes [16].

The combination of coagulation–flocculation with a magnetic separation process offers a useful approach to enhance the separation efficiency. Compared with conventional coagulation–flocculation, magnetic flocculation shows advantages such as high removal efficiency, fast separation speed, wide application scope, and low energy consumption [17]. Conventional magnetic flocculation typically employs an adhesion-based strategy, where colloidal particles are first combined with flocculants and then attached to magnetic seeds to facilitate sedimentation [17,18]. Recent research has increasingly focused on assembling composite flocculants by integrating polymer-based flocculants or surfactants onto the surface of magnetic nanoparticles [19,20,21,22]. The selection of an appropriate surface modifier has been shown to be key in enhancing the interaction between magnetic flocculants and colloidal particles, thereby improving the efficiency of the flocculation process. Leshuk et al. [23] demonstrated that magnetic polymer-based flocculants could be used as a general platform for recovery and recycling of a wide variety of functional nanoparticles. A key innovation in this approach is the reusability of both the nanoparticles and the magnetic flocculant. This makes magnetic flocculants not only a novel method for nanoparticle separation but also an environmentally sustainable alternative to conventional coagulation–flocculation processes, providing a mechanism for the recovery and reuse of polymer additives.

In this work, we developed novel magnetic nanoflocculants using magnetic nanoparticles coated with alternating layers of biopolymers. Two magnetic nanoflocculants were synthesized: MMCA5, by combining chitosan with alginate, and MMCB5, by combining chitosan with BBS. The physical and chemical characterization of the synthesized nanomaterials was performed, followed by their evaluation as magnetic flocculants for the recovery of AgNPs from water. To optimize the process, a response surface methodology based on the Doehlert design was employed, allowing for the assessment of the effects of AgNPs concentration, flocculant concentration, and pH on the AgNPs removal efficiency. The potential application of chitosan, alginate, and BBS in nanoparticle recovery presents innovative opportunities for the development of new nanomaterials, offering an economically sustainable approach to waste valorization.

## 2. Material and Methods

### 2.1. Reagents

Iron (III) chloride hexahydrate (FeCl_3_×6H_2_O, purity > 99%, Germany), trisodium citrate anhydrous (Austria), and polysorbate 20 (TWEEN 20 for molecular biology, Argentina) were purchased from Merk (Darmstadt, Germany). Ethylene glycol (Germany) was purchased from Riedel-de Haën (Darmstadt, Germany). Sodium acetate trihydrate (purity > 99%, USA) was purchased from Fluka Chemika (St. Louis, MO, USA). Tetraethoxylsilane (TEOS, Seelze, Germany), polyethylene glycol (USA), ammonium hydroxide (USA), chitosan (Iceland), alginic acid sodium salt from brown algae (USA), silver nitrate (ACS reagent, ≥99.0%, USA), and sodium dodecylbenzene sulfonate (LAS, USA) were purchased from Sigma-Aldrich (St. Louis, MO, USA). Sodium borohydride was purchased from Biopack (Buenos Aires, Argentina). Humic Acid (Leonardite) was obtained from the International Humic Substance Society (IHSS, Denver, CO, USA). All reagents were used without further purification. The BBS employed in this work was obtained following a procedure detailed elsewhere [24]. Briefly, BBS is derived from gardening-park trimming residues matured for 230 days and digested for 4 h at 60 °C under basic conditions with a 4 V/w water/solid ratio. The resulting suspension was centrifuged for 20 min, and the supernatant was flown through an ultrafiltration membrane (5 kD molecular weight cut-off). The retentate was dried to yield the final BBS product. Chemical composition of BBS is detailed in Table S1, in Supplementary Materials. All aqueous solutions were prepared using ultrapure water.

### 2.2. Synthesis of Magnetic Nanoflocculants (MNFs)

Magnetic iron oxide nanoparticles (MNPs) were synthesized using a modified technique from the literature [25]. Concisely, 1.35 g of FeCl_3_×6H_2_O, 3.6 g of sodium acetate trihydrate, 0.02 g of trisodium citrate, 2 mL of polyethylene glycol, and 40 mL of ethylene glycol were introduced into a Teflon container and homogenized for 30 min with a magnetic stirrer at 500 rpm. The Teflon container was then placed into a stainless-steel autoclave reactor and kept at 200 °C for 24 h. The resulting solid phase was magnetically separated and washed thrice with water, ethanol, and isopropanol, respectively. Subsequently, the as-prepared MNPs were coated with a silica layer: 2.9 g of MNPs were dispersed in 720 mL of an ethanol/water 4:1 solution, and left in agitation at 700 rpm for 20 min. Next, 7.2 mL of NH_4_OH (30%) was added, followed by the dropwise addition of 5.7 mL TEOS. The system was heated at 65 °C for 24 h under magnetic stirring at 700 rpm. The gray precipitate was magnetically separated and washed thrice with the ethanol/water solution. The obtained silica-coated MNPs were utilized as magnetic cores in the subsequent polymer deposition, in a layer-by-layer (LbL) protocol. Two different nanoflocculants were synthesized, based on the combination of five alternating layers of chitosan-alginate (MMCA5) and chitosan-BBS (MMCB5). In both materials, the first and the last layer were chitosan. For deposition of each layer, 100 mL of biopolymer solution was added dropwise to 100 mL of silica-coated iron oxide nanoparticles water dispersion (10 g L^−1^) under vigorous stirring using a paddle stirrer (800 rpm). The chitosan solution consisted of 2 g L^−1^ chitosan, 1 M NaCl, and 2 %wt acetic acid. The alginate solution consisted of 2 g L^−1^ alginate and 1 M NaCl. The BBS solution consisted of 3.6 g L^−1^ BBS and 0.5 M NaCl. The pH of all solutions was adjusted to 5 with NaOH or HCl solutions. Later on, the dispersion was gently stirred for 20 min (300 rpm). The obtained precipitates were washed four times with water, to ensure the removal of excess biopolymers that are not attached to the magnetic flocculant surface.

### 2.3. Synthesis of Silver Nanoparticles (AgNPs)

Two syntheses of AgNPs based on silver salts reduction were used, resulting in AgNPs with different sizes. The first AgNPs synthesis (AgNP1) was performed adapting a procedure described elsewhere [26,27]. A 50 mL solution of 1mM AgNO_3_ was prepared using ultrapure water, and placed in a thermostatic bath at 100 °C. After 5 min of stirring at 500 rpm, 5 mL of 1 %wt sodium citrate solution was added dropwise. The mixture was kept under vigorous stirring (800 rpm) and heating for 30 min. After cooling at room temperature, the suspension was stored in the dark at 4 °C. The second synthesis (AgNP2) was carried out according to the procedure reported by Quintero-Quiroz et al. with modifications [28]. A 5 mL aliquot of sodium citrate solution 0.05 M and a 5 mL aliquot of AgNO_3_ 0.05 M were added to 185 mL of water placed in a thermostatic bath at 6–10 °C. The solution was mixed for 3 min at 800 rpm. Later, 5 mL of 0.05 M NaBH_4_ was added dropwise. Immediately, after the addition of NaBH_4_, the dispersion pH was adjusted to 3 using a 0.01 M HCl solution, to consume the excess of NaBH_4_, and then was adjusted to 10 with a 0.01 M NaOH solution, to ensure the proper storage condition. Finally, the as-obtained AgNP2 was stored in the dark at 4 °C. In both syntheses, we obtained Ag multiple twinned nanoparticles. AgNP1 displayed a heterogeneous distribution, with a mixture of spherical, triangular, and cylindrical morphologies. The average sizes were 40 nm in diameter for spherical nanoparticles, 47 nm in height for triangular prismatic nanoparticles, and 90 nm in length for rice-shaped nanoparticles. In contrast, AgNP2 consisted of spherical nanoparticles with an average size of 10 nm (Figure S1 in Supplementary Materials). AgNP1 and AgNP2 present negative zeta potential values at pH 5, 7, and 9 (Table S2 in Supplementary Materials).

### 2.4. Characterization Techniques

ATR-Fourier transform infrared (FTIR) spectra were measured by using a Spectrum 100 instrument (PerkinElemer, Waltham, MA, USA) in the attenuated total reflectance (ATR) mode with a diamond crystal in the range of 600–4000 cm^−1^. X-ray diffraction (XRD) patterns were performed on a X’Pert PRO MPD diffractometer from PANalytical equipped with Cu anode (45 kV, 40 mA). The XRD patterns were recorded in the range of 2θ value between 10° and 80°. Data management was performed using X’Pert HighScore software 1.0c. N_2_ adsorption–desorption isotherms at 77 K were obtained using a gas adsorption apparatus (ASAP 2020, Micromeritics). Specific surface areas (SSA) were calculated using the Brunauer–Emmett–Teller (BET) model. Thermogravimetric analyses (TGA) were performed with a Q500 Thermogravimetric Analyzer (TA Instruments, New Castle, DE, USA). The analyses were carried out by heating samples at a rate of 10 °C min^−1^ from room temperature to 800 °C in an oxygen atmosphere. Transmission electron microscopy (TEM) images were recorded with a JEOL 2010 instrument equipped with a LaB6 filament and acceleration voltage of 200 kV and a Tecnai F20 G^2^ operated at 200 kV. Samples were deposited onto holey carbon-coated copper grids and onto lacey carbon films. A Malvern Zetasizer Nano ZS90 instrument (Malvern) was employed in the Z potential measurements. Nanoparticles dispersion in a 0.01 M NaCl solution was sonicated for 5 min before the analysis. Magnetic properties were recorded by using a LakeShore 7300 vibrating sample magnetometer. Magnetization curves were registered at 300 K with a magnetic field cycled between −19,000 and 19,000 G.

### 2.5. Experimental Procedure

The MNFs were evaluated for their performance toward AgNPs recovery from water. The experiences were conducted in an orbital shaker (Vicking, shaker pro model 6004), where AgNPs aqueous dispersion was mixed with nanoflocculants dispersion (i.e., MMCA5 or MMCB5) with a final volume of 10 mL. The operating conditions comprised a rapid mixing for 2 min at 125 rpm and a slow mixing step for 20 min at 90 rpm. Subsequently, the flocs were magnetically separated, and an aliquot of the supernatant was withdrawn and analyzed by UV–vis spectroscopy at 420 nm (PG Instruments, model 760 UV) to determine the AgNPs concentration (Figure S2 in Supplementary Materials). The removal performance for each material was determined using the removal efficiency percentage (%RE):

(1) $$\%RE=\frac{initial\;AgNPs\;concentration-final\;AgNPs\;concentration}{initial\;AgNPs\;concentration}\times 100$$

Experiments were also carried out in the presence of certain substances, to evaluate their influence in AgNPs removal: NaCl (0.01 M), NaNO_3_ (0.01 M), Na_2_SO_4_ (0.01 M), humic acids (5–10 mg L^−1^), LAS (10–20 mg L^−1^), and TWEEN 20 (10–20 mg L^−1^).

### 2.6. Surface Response Methodology

In the present study, an experimental design methodology based on a Doehlert matrix was employed to analyze the effect of three factors: AgNPs concentration (X_1_, mg L^−1^), nanoflocculant concentration (X_2_, mg L^−1^), and pH (X_3_) on the efficient removal of AgNPs (%RE). In total, 15 experiments were carried out for each nanoflocculant, including three repetitions at the center point. Response surface model fitting was performed with NemrodW software 1.0, using the least squares method. The %RE of AgNPs was used as a response. A second-degree polynomial was used to correlate the response and the three independent variables:

(2) $$Y=b_{0}+\sum b_{i}X_{i}+\sum b_{ii}X_{i}^{2}+\sum b_{ij}X_{i}X_{j}$$

Y represents the %RE of AgNPs, $b_{0},b_{i},b_{ii},b_{ij}$ the constant coefficients, and $X_{i}$ the level of the selected factors. Analysis of variance (ANOVA) and a response surface diagram were performed. The quality of the model fit and the predictive capacity were expressed by the coefficient of determination (R^2^).

### 2.7. Reuse Experiments and Nanoflocculant Regeneration

To evaluate the release of recovered AgNPs and the potential reuse of the nanoflocculants, multiple cycles of flocculation and deflocculation experiments were performed. The first step in the process was the previously mentioned flocculation experiment and the analysis of AgNPs removal, under the experimental conditions: [AgNPs] = 13.5 mg L^−1^, [MNF] = 1000 mg L^−1^, pH = 5. Subsequently, the supernatant was removed from the vial, and the remaining flocs were washed with 10 mL of pH 10 adjusted water, sonicated for 5 min and magnetically separated for 30 min. The concentration of the released AgNPs was determined. The regenerated MNFs were then reused for subsequent flocculation experiments. This cycle of flocculation, regeneration and reuse processes was repeated until the removal efficiency of the MNFs decreased to less than 40%.

## 3. Results and Discussion

### 3.1. Characterization

**Figure 1:**
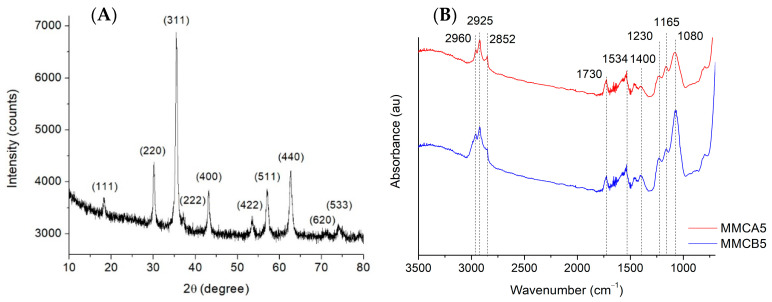
(**A**) XRD diffraction patterns of the silica-coated magnetic iron oxide nanoparticles. (**B**) ATR-FTIR spectra of MNFs.

To prepare the MNFs, silica-coated magnetic iron oxide nanoparticles were initially synthesized where the silica shell serves as a protective layer against the oxidation and dissolution of the magnetic core during the flocculation process. The crystallographic phases and the efficiency of the silica coating on these nanoparticles were analyzed. Figure 1A shows the X-ray diffraction pattern of silica-coated magnetic iron oxide nanoparticles. All observed reflections can be associated with magnetic iron oxide phases. In particular, peak positions at 2θ = 18.3, 30.07, 35.41, 37.04, 43.04, 53.4, 56.91, 62.5, 70.9, and 73.9, can be associated with (111), (220), (311), (222), (400), (422), (511), (440), (620), and (533) crystalline planes of the magnetite phase (Card number 01-076-1849, ICCD Data base). However, the presence of the maghemite (γ-Fe_2_O_3_) phase cannot be discarded, since magnetite and maghemite have the same cubic structure and almost identical lattice parameters [29]. To determine whether the silica coating procedure was complete and stable, two samples with different coating times (8 and 24 h) and bare iron oxide nanoparticles were subjected to acid treatment (see Supplementary Materials for further details). The iron solubilization rate in acidic conditions can be regarded as an indirect measurement of the degree of iron oxide coating and the nature of the silica coating [30]. Figure S3 in Supplementary Materials shows that both 8 h and 24 h of time for the deposition of the silica layer seem to be efficient in protecting the magnetic iron oxide cores from the acidic media, although it is observed that at 24 h the dissolution of iron was delayed for a longer time. Thus, the most extensive coating procedure (24 h) was selected for the syntheses of MNFs.

**Figure 2:**
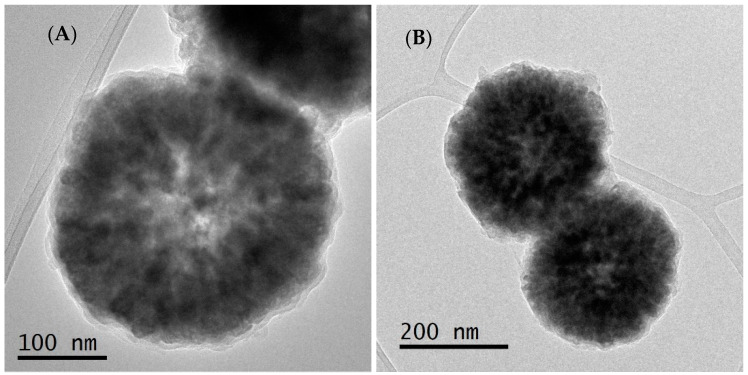
TEM images of MNFs: (**A**) MMCA5 and (**B**) MMCB5.

**Figure 3:**
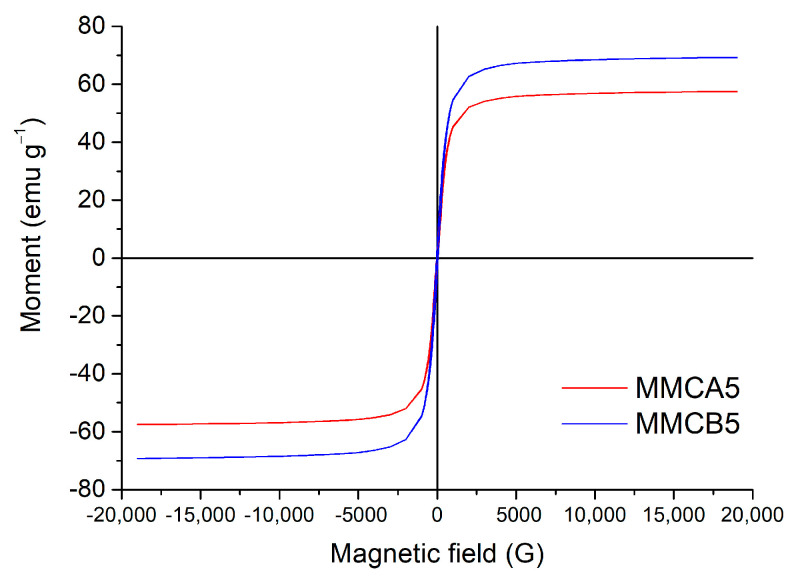
Magnetization curves of MNFs.

MMCA5 and MMCB5 were then obtained via biopolymers deposition using the LbL method onto silica-coated magnetic iron oxide nanoparticles according to the procedure described in Section 2.2. The presence of biopolymer layers on MMCA5 and MMCB5 was confirmed by FTIR analysis (Figure 1B). The absorption bands at 2960 cm^−1^, 2925 cm^−1^, 2852 cm^−1^ (C-H stretching), 1730 cm^−1^ (C=O stretching), 1534 cm^−1^ (N-H flexion), 1400 cm^−1^ (COO symmetrical stretching), 1230 cm^−1^ (C-N stretching), and 1165 cm^−1^ (C-O bridge- stretching) could be associated with the characteristic groups of chitosan, alginate, and BBS [15,31]. The presence of the silica shell is evidenced by a 1080 cm^−1^ band (Si-O-Si stretching) [16]. The TEM images (Figure 2) show that MMCA5 and MMCB5 have spherical morphology and are approximately 200 nm in diameter. The magnetic cores are composed of self-assembled small primary nanocrystals of iron oxide of approximately 10 nm in diameter. The amorphous outer layer that surrounds the clusters confirms the presence of silica. Figure 3 displays the magnetization curves obtained for MMCB5 and MMCA5 at 300 K. All samples exhibited soft ferromagnetic behavior with low coercivity and remanence (Table S3, in Supplementary Materials). Saturation magnetization for MMCB5 and MMCA5 was 69.3 and 57.5 emu g^−1^, respectively, which are slightly lower than that pure magnetite (79.5 emu g^−1^) [32]. These results demonstrate a strong magnetic response and a high potential of MNFs to be effectively magnetically separated from the medium.

**Figure 4:**
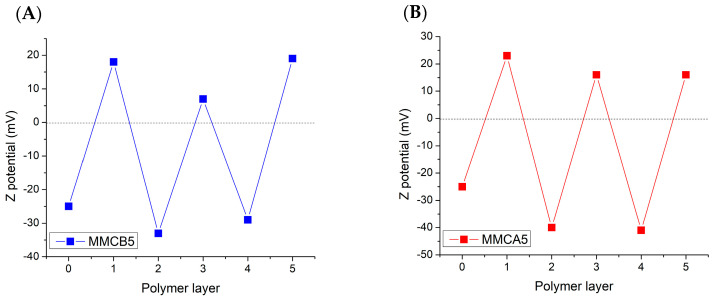
Zeta potential as a function of the biopolymer layer number for each MNFs. (**A**) MMCB5: the positive potentials represent chitosan coating, while the negative potentials represent BBS coating. (**B**) MMCA5: the positive potentials represent chitosan coating, while the negative potentials represent alginate coating. Nanoflocculants dispersed in 0.01 M NaCl solution at pH 5.

Table S4 in Supplementary Materials displays the mass loss for both samples at each stage of the LbL MNF synthesis measured via TGA. The increase in weight loss from the first chitosan layer deposition to MMCA5 and MMCB5 corroborates the successive deposition of biopolymers in the synthesis protocol. Furthermore, the low mass loss values indicate that the polymer coatings possess low film thickness. Additionally, to confirm the polymer deposition throughout the LbL coating process, zeta potential measurements were also performed during the synthesis procedure (Figure 4). The alternating positive and negative zeta potential values with the addition of the biopolymers confirm the successful deposition of chitosan, alginate, and BBS. Specifically, after the addition of negatively charged polymers such as alginate and BBS, negative zeta potential values were observed, indicating the successful deposition of these polymers onto the nanoparticle surface. Similarly, after the addition of chitosan, a positively charged polymer at the working pH, positive zeta potential values were observed, confirming the successful deposition of this polymer as well. Furthermore, zeta potential of both MNFs at pH 5, 7, and 9 are shown in Table S5 in Supplementary Materials. Positive zeta potential values were observed at pH 5, whereas at pH 7 and 9, both MNFs exhibited negative values.

### 3.2. AgNPs Removal Using MMCA5 and MMCB5

**Figure 5:**
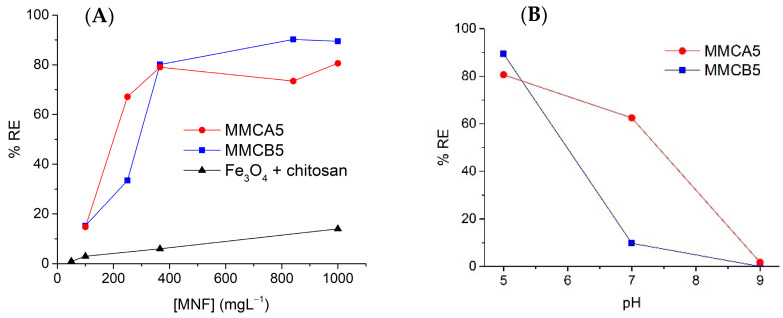
(**A**) Effect of MNF dosage on AgNPs removal. [AgNP1] = 13.5 mg L^−1^, [chitosan] = 50 mg L^−1^, pH = 5. (**B**) Effect of pH on AgNPs removal. [AgNP1] = 13.5 mg L^−1^, [MNF] = 1000 mg L^−1^.

MMCA5 and MMCB5 were used as a magnetic flocculant to remove AgNPs from waters. Initially, AgNP1 samples were used as a source of AgNPs in the experiments of this section, because this sample is more heterogenous in morphology and has a wider size dispersion. As a first approach, single-factor experiments were carried out to analyze the effect of MNFs dosage on the RE% (Figure 5A). At low dosages, the removal of AgNPs increases significantly with the addition of flocculants, reaching nearly 80% removal at a dosage of 400 mg L^−1^. Beyond this point, the AgNPs removal slightly increased when increasing the dosage of MNF. Both MNFs exhibited a similar trend; however, MMCB5 demonstrated superior performance, achieving 90% AgNPs removal at a dosage of 800 mg L^−1^. Throughout the wide range of flocculant concentrations tested, no overdosing effects were observed, highlighting the advantage of MNF over conventional flocculants. Additionally, the effect of pH on %RE at the highest MNF dosage was analyzed (Figure 5B). As shown, pH plays a critical role in AgNPs removal. The highest %RE values were obtained at pH 5, followed by a sharp decrease at pH 9. Interestingly, MMCA5 exhibited good %RE even at pH 7.

To evaluate the individual effects of chitosan and magnetic iron oxide nanoparticles on AgNPs removal, control experiments were conducted. In these experiments, a dispersion of AgNPs was exposed to varying concentrations of chitosan and magnetic iron oxide nanoparticles under the same stirring conditions used in the MNF experiments. Table S6 in Supplementary Materials presents the conditions used and the %RE achieved for each case. For comparison purposes, the AgNPs removal results using free chitosan (50 mg L^−1^) and varying amounts of magnetic iron oxide nanoparticles are shown in Figure 5. As shown, none of the conditions resulted in the removal of more than 15% of the AgNPs, indicating that the individual components (i.e., biopolymer and magnetic iron oxide nanoparticles) do not produce the same flocculant effect as the MNFs. These results clearly demonstrate that the integration of the biopolymers on the surface of magnetic nanoparticles is crucial for their effectiveness in separating AgNPs from water.

The influence and the interaction of MNF dosage, AgNPs concentration and pH were evaluated via an experimental design methodology based on a Doehlert matrix. The experimental conditions and the results are displayed in Table 1. The variables ranges were 7–20 mg L^−1^ (AgNP1 concentration), 50–1000 mg L^−1^ (MNF concentration), and 5–9 (pH).

The results of the ANOVA test for AgNPs removal efficiency and model coefficients are shown in Tables S7 and S8 in Supplementary Materials, respectively. For MMCB5, all terms are statistically significant (*p*-value < 0.05), except for the independent term of the polynomial (b_0_). In contrast, for MMCA5, the terms associated with AgNPs concentration show no significant effect on the RE%. In both models, pH had the most important effect on AgNP1 removal, which supports the hypothesis of electrostatic interaction between AgNP1 and the MNF. The resulting polynomial equations, including only the significant parameters and interactions, are presented below:

MMCA5 Polynomial:

% RE = 29.66 + 24.19[MNFs] − 37.29[pH] + 9.69[MNFs] ^2^ − 8.81[MNFs].[pH]

MMCB5 Polynomial:

% RE = −1.11[AgNPs] + 7.27[MNFs] − 41.32[pH] + 4.24[AgNPs]^2^ + 7.67[MNFs]^2^ + 47.46[pH]^2^ + 10.22[AgNPs].[MNFs] + 13.09 [AgNPs].[pH] − 22.75[MNFs].[pH]

The R^2^ adjusted values for each MNF model were 0.90 and 0.92 for MMCB5 and MMCA5, respectively. These R^2^_adj_ values are indicative of the percentage of variance explained by the model [33]. The correlation between experimental and predicted responses are presented in Figure S4 in Supplementary Materials. Determination coefficients (R^2^) greater than 0.9 for each model confirm the correct prediction of the statistical models.

**Figure 6:**
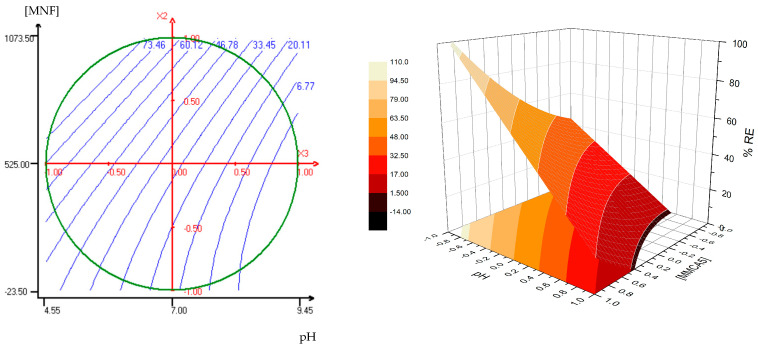
Response surface plot and the isoresponse curve (at [AgNP1] = 13.5 mg L^−1^) for MMCA5.

**Figure 7:**
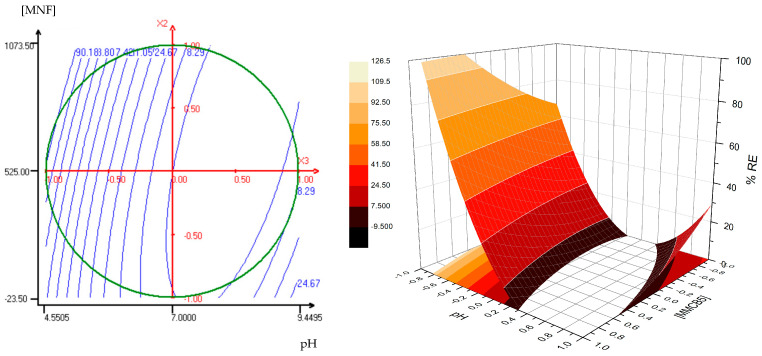
Response surface plot and the isoresponse curve (at [AgNP1] = 13.5 mg L^−1^) for MMCB5.

Figure 6 and Figure 7 display the response surface plots and isoresponse curves for MMCA5 and MMCB5, respectively. No maximum was found within the studied conditions and no re-stabilization effect of AgNPs was observed at high flocculation concentration for both MNFs. For MMCA5, an increase in removal efficiency is observed with higher concentrations of flocculant, reaching a maximum removal of approximately 80% at lower pH values. For MMCB5, a similar trend is observed, with high RE% predicted at low pH values and high MNF concentrations. In general, at pH 5 and MNF concentrations above 600 mg L^−1^, over 90% AgNPs removal is expected. Additionally, an artifact appears at high pH and low MNF concentrations, where no removal was observed experimentally.

**Figure 8:**
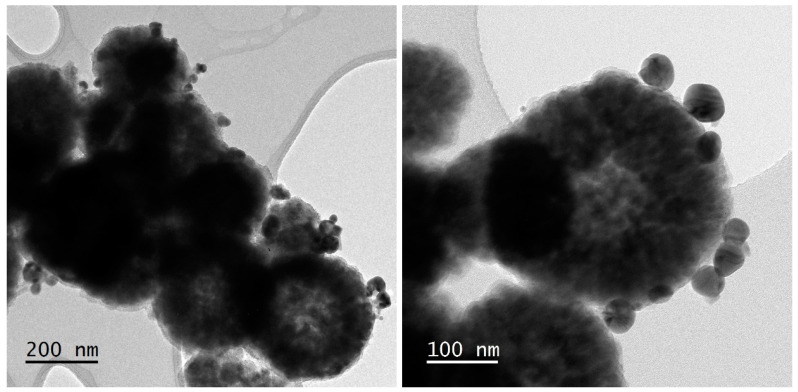
TEM images of AgNPs after the magnetic flocculation process using MMCB5.

Comparing both surfaces, MMCA5 demonstrates a wider range of applicability across the studied pH values, whereas MMCB5 achieves higher AgNPs removal efficiency. This pH effect was also evidenced in the single factor experiments (Figure 5B). The superior performance of MNFs under acidic conditions can be attributed to the surface charges of AgNPs and the synthesized flocculants. The zeta potential of AgNPs ranged from −36 mV to −43 mV in the studied pH levels, whereas MNFs exhibited a positive zeta potential at pH 5 and a negative zeta potential at pH 7 and 9 (Tables S4 and S5 in Supplementary Materials). Consequently, the interaction between these materials is likely electrostatic. These results suggest that the primary mechanism for AgNPs removal is charge neutralization, wherein the positively charged chitosan layer on the MNFs interacts with the negatively charged surface of the AgNPs. Microscopic observation of the interparticle interaction between AgNPs and MMCB5 shows that AgNPs are attached to the surface of the MNF and are also surrounded by a floc of MNF particles (Figure 8). Thus, the removal of AgNPs by the MNF likely occurs through electrostatic interactions between the AgNPs and nanoflocculants, followed by the adsorption of AgNPs onto the MNF surface and the formation of flocs due to reduced electrostatic repulsion. Finally, the sedimentation of these flocs is accelerated by the applied magnetic field.

**Figure 9:**
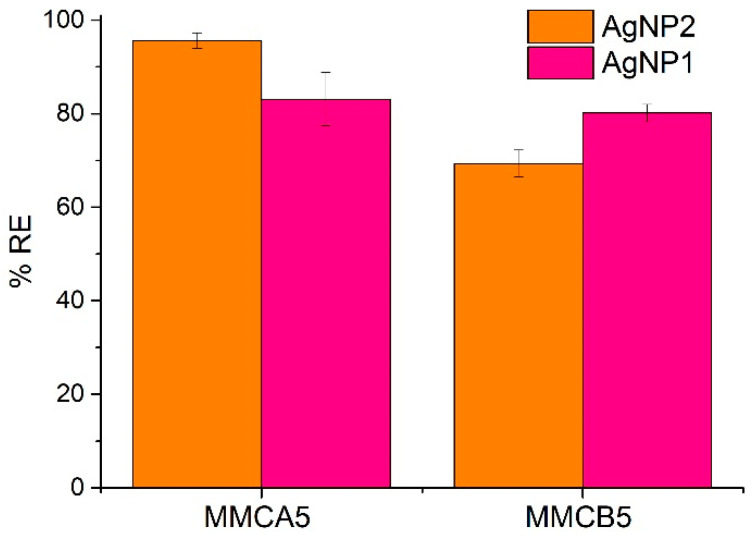
MNF performance in removing AgNP1 and AgNP2. [MNF] = 366 mg L^−1^, [AgNPs] = 13.5 mg L^−1^, pH = 5.

The effect of HA, NaCl, NaNO_3_, Na_2_SO_4_, and two surfactants, anionic sodium-dodecylbenzene-sulfonate (LAS) and non-ionic polyoxyethylene (20) sorbitan monolaurate, (TWEEN 20) on MNF performance was evaluated (Table 2). These substances were selected because they can be present in wastewater and natural waters. In particular, LAS and TWEEN 20 are commonly found in different types of wastewaters [34,35]. The results indicate that HA and LAS significantly reduce MNF performance, leading to a noticeable decrease in %RE, while coexisting anions (e.g., Cl^−^, NO_3_^−^, and SO_4_^2−^) and TWEEN 20 have minimal to no effect on AgNPs removal efficiency. In particular, the presence of HA leads to a substantial decrease in %RE, with removal efficiency dropping to approximately 10% at a HA concentration of 10 mg L^−1^. These results suggest that HA as well as LAS may screen the positive charges on chitosan, hindering its interaction with AgNPs. Zeta potential measurements of MNF aqueous dispersions with HA, LAS, and TWEEN 20 (Table S9 in Supplementary Materials) confirmed the interaction of the anionic substances with the MNF surfaces. This is evidenced by a significant decrease in zeta potential values in the presence of LAS and the transition to negative zeta potential values in the presence of HA. In the case of TWEEN 20, almost no change in the zeta potential values were observed. Moreover, the UV-Vis spectra of AgNPs dispersed in an aqueous solution in the presence of TWEEN 20 (Figure S5 in Supplementary Materials) indicate that TWEEN 20 neither affects nor destabilizes the AgNPs dispersion. This confirms that the high removal of AgNPs observed in the presence of TWEEN 20 is attributed to the action of the nanoflocculant. These results reinforce the suggestion that electrostatic interactions played an important role in the process of AgNPs removal. The same mechanism was observed in the removal of TiO_2_ nanoparticles and kaolin suspensions via magnetic coagulation and flocculation processes [18,23]. Additionally, both MNFs were tested for their capacity to remove smaller AgNPs (AgNP2) from suspension (Figure 9). Under the selected conditions, no significant differences or clear trends were observed in the removal of the different AgNPs batch samples, suggesting that both MNFs are effective in removing AgNPs with diameters ranging from 10 nm to 90 nm

**Figure 10:**
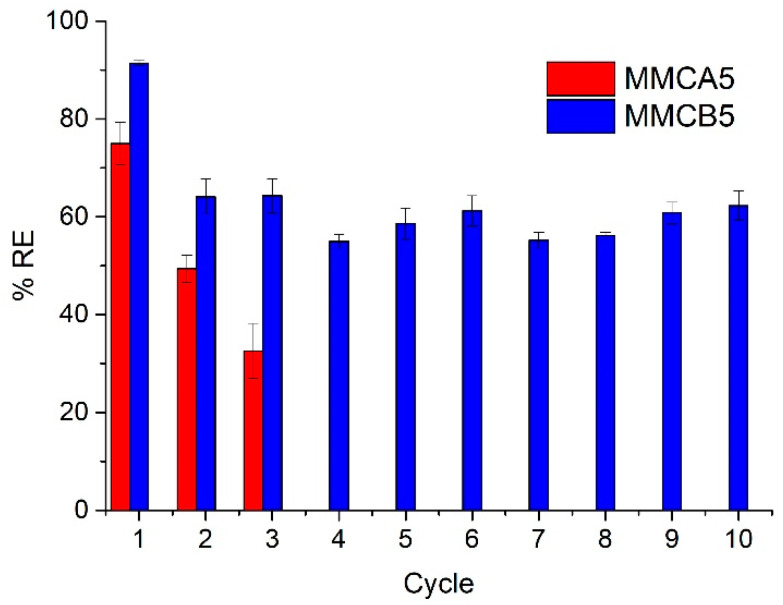
Effect of recycling of MNFs on the AgNP1 removal efficiency after various cycles of use. [MNF] = 1000 mg L^−1^, [AgNP1] = 13.5 mg L^−1^, pH = 5.

Figure 10 shows the results of the recycling experiments conducted on the MNFs. The removal efficiency of AgNP1 decreased after the first cycle. This phenomenon may be attributed to the leaching of biopolymers during the washing processes of the MNF. Among the tested materials, MMCB5 demonstrated the highest performance, successfully removing over 50% of the initial AgNPs across ten consecutive cycles. On the other hand, MMCA5 exhibited a gradual decrease in AgNP1 removal efficiency with each cycle, achieving approximately 35% removal of AgNPs concentration by the third experiment.

The good performance of MMCB5 suggests that the external chitosan shell may be adsorbed more effectively into the MNF surface due to the presence of BBS, resulting in increased stability during the washing process at pH 10. The complex structure of BBS, which includes various chemical groups (e.g., aromatic, aliphatic, phenolic, and carboxylic groups), likely contributes to the formation of a more stable flocculant following the LbL deposition of the polymers.

## 4. Conclusions

This study presents a novel approach to the removal and recovery of AgNPs from water using sustainable magnetic nanoflocculants. The MNFs, synthesized by coating magnetic iron oxide nanoparticles with alternating layers of biopolymers (chitosan-alginate, and chitosan-bio-based substances from urban waste), demonstrated significant potential for AgNPs separation. The results indicated that the AgNPs removal efficiency is influenced by various factors, including pH, MNF concentration, and AgNPs concentration, with optimal removal achieved at low pH and high MNF concentrations. Among the tested MNFs, MMCB5, incorporating chitosan and bio-based substances, exhibited the highest AgNPs removal efficiency, achieving up to 90% removal at pH 5 and 600 mg L^−1^ MNF concentration. Charge neutralization was identified as the primary mechanism responsible for the removal of AgNPs. These magnetic flocculants did not show the overdosing effects typically associated with conventional flocculants. The study also highlights the robust performance of the MNFs in removing AgNPs, even in the presence of coexisting anions, such as Cl^−^, NO_3_^−^, SO_4_^2−^, and non-ionic surfactants. Furthermore, the recycling experiments demonstrated the reusability of the MNFs, with MMCB5 maintaining 50% of its initial removal efficiency after ten cycles. The superior performance achieved with MMCB5 paves the way for the application of bio-based substances as substances that can be utilized in the design of new materials for environmental remediation. Overall, the findings suggest that the developed MNFs provide an effective, eco-friendly, and economically sustainable solution for AgNPs recovery from aqueous systems, offering promising applications in water treatment and nanoparticle contamination remediation.

## Figures and Tables

**Table 1:** Experimental conditions used for the response surface methodology analysis. The data given in the last two columns correspond to the removal percentage of AgNPs obtained by each material.

Exp	Experimental Conditions			MMCB5	MMCA5
	AgNP1 (mg L^−1^)	MNF (mg L^−1^)	pH	RE (%)	RE (%)
1	20.0	525	7	8.8	30.6
2	7.0	525	7	0	36.1
3	16.7	1000	7	14.4	60.3
4	10.2	50	7	8.5	12.1
5	16.7	50	7	0	13.9
6	10.2	1000	7	5.1	65.1
7	16.7	683	9	0	2.6
8	10.2	366	5	69.9	59.8
9	16.7	366	5	42.6	52.9
10	13.5	841	5	90.2	73.5
11	10.2	683	9	0	0.8
12	13.5	208	9	0	0
13	13.5	525	7	0.1	28.9
14	13.5	525	7	0	28.9
15	13.5	525	7	0.4	31.3

**Table 2:** Results of the effect of HA, NaCl, NaNO_3_, Na_2_SO_4_, and surfactants concentration on AgNP1 removal.

Material	[HA] (mg L^−1^)	[LAS] (mg L^−1^)	[TWEEN 20] (mg L^−1^)	[NaCl] (M)	[NaNO_3_] (M)	[Na_2_SO_4_] (M)	% RE *
MMCA5	-	-	-	-	-	-	79
	5	-	-	-	-	-	30
	10	-	-	-	-	-	11
	-	10	-	-	-	-	45
	-	20	-	-	-	-	43
	-	-	10	-	-	-	95
	-	-	20	-	-	-	94
	-	-	-	0.01	-	-	90
	-	-	-	-	0.01	-	86
	-	-	-	-	-	0.01	80
MMCB5	-	-	-	-	-	-	80
	5	-	-	-	-	-	17
	10	-	-	-	-	-	9
	-	10	-	-	-	-	51
	-	20	-	-	-	-	31
	-	-	10	-	-	-	75
	-	-	20	-	-	-	80
	-	-	-	0.01	-	-	81
	-	-	-	-	0.01	-	76
	-	-	-	-	-	0.01	70

[MNF] = 366 mg L^−1^, [AgNP1] = 13.5 mg L^−1^, pH = 5. * RSD < 10%.

## Data Availability

Data is contained within the article.

## Supplementary Materials

The following supporting information can be downloaded at https://www.mdpi.com/article/10.3390/polym17050650/s1: Figure S1. TEM images and size distribution for AgNP1 and AgNP2. Figure S2. (A) UV/Vis spectra of AgNP1 aqueous suspensions and (B) calibration curves prepared from the spectra at 420 nm. Figure S3. Acidic dissolution of bare MNPs and silica-coated MNPs. Figure S4. Correlation of experimental and predicted responses. Figure S5. UV-Vis absorption spectra of AgNPs, tween 20, and AgNPs + tween 20. Table S1. BBS chemical composition and functional groups (a). Table S2. Magnetic properties for MMCB5 and MMCA5. Table S3. Calculated weight loss after deposition of each polymer layer during the synthesis of MMCB5 and MMCA5 from TGA analysis. Table S4. Zeta potential measurements of MMCB5 and MMCA5 at pH 5, 7, and 9. Table S5. Zeta potential measurements of AgNP1 and AgNP2 at pH 5, 7, and 9. Table S6. Control experiments. Results of magnetic iron oxide nanoparticles and chitosan in AgNPs removal. Table S7. Analysis of variance (ANOVA) for %RE of MNFs. Table S8. Analysis of variance (ANOVA) for the selected variables. Table S9. Zeta potential measurements of MNFs dispersion at pH 5 in the presence of different additives.

## References

[1] Temizel-Sekeryan S., Hicks A.L. (2020). Global Environmental Impacts of Silver Nanoparticle Production Methods Supported by Life Cycle Assessment. Resour. Conserv. Recycl..

[2] StatNano Nanotechnology Products Database (NPD) [WWW Document]. http://Product.Statnano.Com/.

[3] McGillicuddy E., Murray I., Kavanagh S., Morrison L., Fogarty A., Cormican M., Dockery P., Prendergast M., Rowan N., Morris D. (2017). Silver Nanoparticles in the Environment: Sources, Detection and Ecotoxicology. Sci. Total Environ..

[4] Yu S.J., Yin Y.G., Liu J.F. (2013). Silver Nanoparticles in the Environment. Environ. Sci. Process. Impacts.

[5] Cleveland D., Long S.E., Pennington P.L., Cooper E., Fulton M.H., Scott G.I., Brewer T., Davis J., Petersen E.J., Wood L. (2012). Pilot Estuarine Mesocosm Study on the Environmental Fate of Silver Nanomaterials Leached from Consumer Products. Sci. Total Environ..

[6] Marambio-Jones C., Hoek E.M.V. (2010). A Review of the Antibacterial Effects of Silver Nanomaterials and Potential Implications for Human Health and the Environment. J. Nanopart. Res..

[7] Fabrega J., Luoma S.N., Tyler C.R., Galloway T.S., Lead J.R. (2011). Silver Nanoparticles: Behaviour and Effects in the Aquatic Environment. Environ. Int..

[8] Mat Lazim Z., Salmiati S., Marpongahtun M., Arman N.Z., Mohd Haniffah M.R., Azman S., Yong E.L., Salim M.R. (2023). Distribution of Silver (Ag) and Silver Nanoparticles (AgNPs) in Aquatic Environment. Water.

[9] Troester M., Brauch H.J., Hofmann T. (2016). Vulnerability of Drinking Water Supplies to Engineered Nanoparticles. Water Res..

[10] Verma A.K., Dash R.R., Bhunia P. (2012). A Review on Chemical Coagulation/Flocculation Technologies for Removal of Colour from Textile Wastewaters. J. Environ. Manag..

[11] Frantz T.S., de Farias B.S., Leite V.R.M., Kessler F., Cadaval T.R.S.A., Pinto L.A.d.A. (2020). Preparation of New Biocoagulants by Shrimp Waste and Its Application in Coagulation-Flocculation Processes. J. Clean. Prod..

[12] Yang R., Li H., Huang M., Yang H., Li A. (2016). A Review on Chitosan-Based Flocculants and Their Applications in Water Treatment. Water Res..

[13] El Foulani A.A., Ounas O., Laabi A., Lekhlif B., Jamal-Eddine J. (2020). Removal of Dissolved and Colloidal Matter from Surface Waters by Composite Flocculant Aluminum Salt-Sodium Alginate. Desalin. Water Treat..

[14] Avetta P., Bella F., Bianco Prevot A., Laurenti E., Montoneri E., Arques A., Carlos L. (2013). Waste Cleaning Waste: Photodegradation of Monochlorophenols in the Presence of Waste-Derived Photosensitizer. ACS Sustain. Chem. Eng..

[15] Magnacca G., Allera A., Montoneri E., Celi L., Benito D.E., Gagliardi L.G., Gonzalez M.C., Mártire D.O., Carlos L. (2014). Novel Magnetite Nanoparticles Coated with Waste-Sourced Biobased Substances as Sustainable and Renewable Adsorbing Materials. ACS Sustain. Chem. Eng..

[16] Peralta M.E., Koffman-Frischknecht A., Moreno M.S., Mártire D.O., Carlos L. (2023). Application of Biobased Substances in the Synthesis of Nanostructured Magnetic Core-Shell Materials. Inorganics.

[17] Wang K., Mao Y., Wang C., Ke Q., Zhao M., Wang Q. (2022). Application of a Combined Response Surface Methodology (RSM)-Artificial Neural Network (ANN) for Multiple Target Optimization and Prediction in a Magnetic Coagulation Process for Secondary Effluent from Municipal Wastewater Treatment Plants. Environ. Sci. Poll. Res..

[18] Liu C., Wang X., Qin L., Li H., Liang W. (2021). Magnetic Coagulation and Flocculation of a Kaolin Suspension Using Fe3O4 Coated with SiO_2_. J. Environ. Chem. Eng..

[19] Wang M., Feng L., Fan X., Li D., Qu W., Jiang S., Li S. (2018). Fabrication of Bifunctional Chitosan-Based Flocculants: Characterization, Assessment of Flocculation, and Sterilization Performance. Materials.

[20] Han S.F., Jin W., Tu R., Gao S.H., Zhou X. (2020). Microalgae Harvesting by Magnetic Flocculation for Biodiesel Production: Current Status and Potential. World J. Microbiol. Biotechnol..

[21] Liu C., Wang X., Du S., Liang W. (2024). Synthesis of Chitosan-Based Grafting Magnetic Flocculants for Flocculation of Kaolin Suspensions. J. Environ. Sci..

[22] Yang J., Wang J., Guo J., Zhang Y., Zhang Z. (2023). Dendrimer Modified Composite Magnetic Nano-Flocculant for Efficient Removal of Graphene Oxide. Sep. Purif. Technol..

[23] Leshuk T., Holmes A.B., Ranatunga D., Chen P.Z., Jiang Y., Gu F. (2018). Magnetic Flocculation for Nanoparticle Separation and Catalyst Recycling. Environ. Sci. Nano..

[24] Nisticò R., Cesano F., Franzoso F., Magnacca G., Scarano D., Funes I.G., Carlos L., Parolo M.E. (2018). From Biowaste to Magnet-Responsive Materials for Water Remediation from Polycyclic Aromatic Hydrocarbons. Chemosphere.

[25] Zhang Y., Xu J., Li Q., Cao D., Li S. (2019). The Effect of the Particle Size and Magnetic Moment of the Fe3O4 Superparamagnetic Beads on the Sensitivity of Biodetection. AIP Adv..

[26] Caresio A. (2021). Materiali Ossidici Funzionalizzati per il Sequestro di Nanoparticelle Metalliche da Matrici Acquose. Master’s Thesis.

[27] Chowdhury S., Yusof F., Faruck M.O., Sulaiman N. (2016). Process Optimization of Silver Nanoparticle Synthesis Using Response Surface Methodology. Procedia Eng..

[28] Quintero-Quiroz C., Acevedo N., Zapata-Giraldo J., Botero L.E., Quintero J., Zárate-Trivinõ D., Saldarriaga J., Pérez V.Z. (2019). Optimization of Silver Nanoparticle Synthesis by Chemical Reduction and Evaluation of Its Antimicrobial and Toxic Activity. Biomater. Res..

[29] Sarveena, Vargas J.M., Shukla D.K., Meneses C.T., Mendoza Zélis P., Singh M., Sharma S.K. (2016). Synthesis, Phase Composition, Mössbauer and Magnetic Characterization of Iron Oxide Nanoparticles. Phys. Chem. Chem. Phys..

[30] Bruce I.J., Taylor J., Todd M., Davies M.J., Borioni E., Sangregorio C., Sen T. (2004). Synthesis, Characterisation and Application of Silica-Magnetite Nanocomposites. J. Magn. Magn. Mater..

[31] Peralta M.E., Nisticò R., Franzoso F., Magnacca G., Fernandez L., Parolo M.E., León E.G., Carlos L. (2019). Highly Efficient Removal of Heavy Metals from Waters by Magnetic Chitosan-Based Composite. Adsorption.

[32] Peralta M.E., Jadhav S.A., Magnacca G., Scalarone D., Mártire D.O., Parolo M.E., Carlos L. (2019). Synthesis and in Vitro Testing of Thermoresponsive Polymer-Grafted Core-Shell Magnetic Mesoporous Silica Nanoparticles for Efficient Controlled and Targeted Drug Delivery. J. Colloid Interface Sci..

[33] Carabajal M., Teglia C.M., Maine M.A., Goicoechea C. (2021). Multivariate Optimization of a Dispersive Liquid-Liquid Microextraction Method for the Determination of Six Antiparasite Drugs in Kennel Effluent Waters by Using Second-Order Chromatographic Data. Talanta.

[34] Selambakkannu S., Othman N.A.F., Bakar K.A., Thailan K.M., Karim Z. (2021). Degradation of Surfactants from Domestic Laundry Effluent by Electron Beam Irradiation. Mater. Today Proc..

[35] Terreros-Mecalco J., Velazquez-Garduño G., Archundia Velarde E.D., Hernández Ruiz P.E. (2024). Effect of Lactose as a Co-Substrate in the Biodegradation of an Anionic Surfactant “LAS” by Anaerobic Digestion. Biocatal. Agric. Biotechnol..

