# Supplementary material for: Removal and Recovery of AgNPs from Water by Sustainable Magnetic Nanoflocculants
Source: Polymers (Basel). 2025 Feb 28;17(5):650. doi: 10.3390/polym17050650 (PMC11902812; doi:10.3390/polym17050650)
Supplement: Supplementary file 1 [file polymers-17-00650-s001.zip › polymers-3446495-supplementary.pdf]

Supplementary Material

Removal and Recovery of AgNPs from Water by Sustainable Magnetic Nanoflocculants

Mariana Ramirez, Eya Ben Khalifa, Giuliana Magnacca, M. Sergio Moreno, María E. Parolo, Luciano Carlos

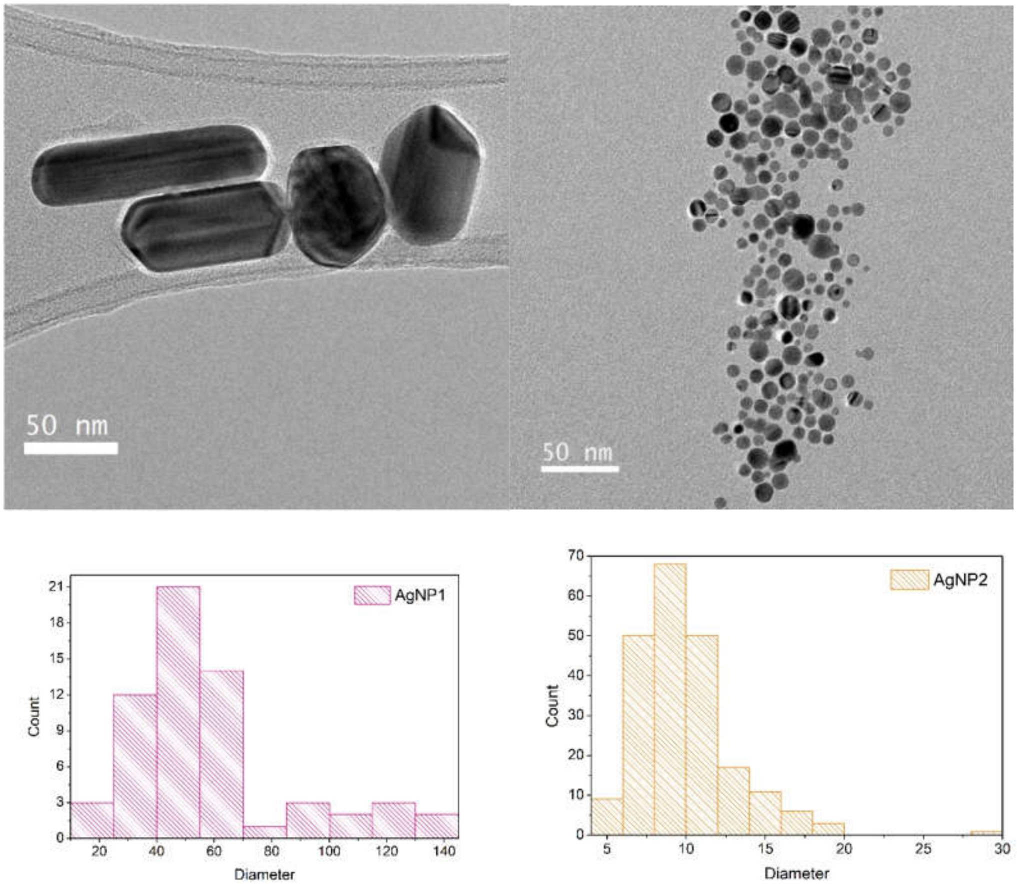

Figure S1. TEM images and size distribution for AgNP1 and AgNP2.

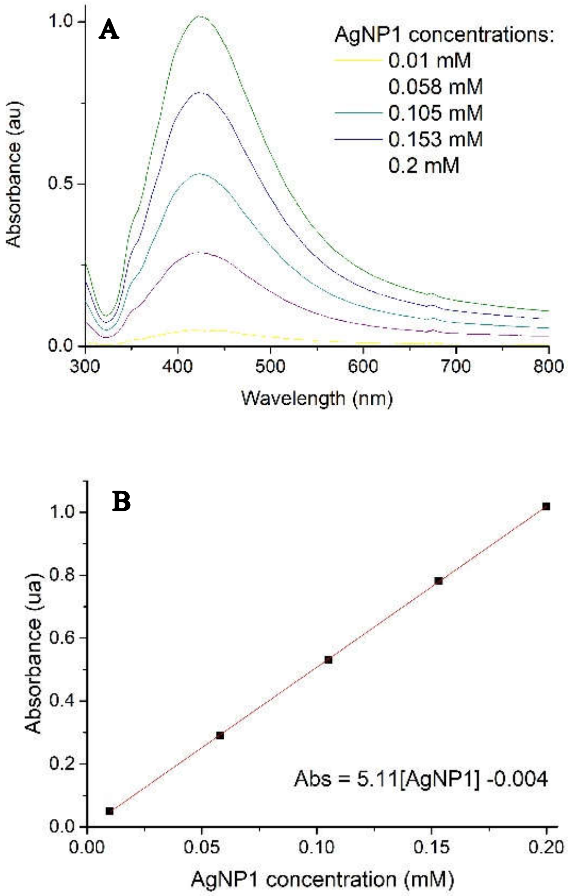

Figure S2. A) UV/Vis spectra of AgNP1 aqueous suspensions and B) calibration curves prepared from the spectra at 420 nm.

Acidic dissolution of silica coating magnetic iron oxide nanoparticles

To determine whether the silica coating procedure was complete and stable, two samples with different coating times (8 and 24 h) and bare magnetic iron oxide nanoparticles (MNPs) were subjected to acid treatment during 24 h. Briefly, 25 g samples of bare MNPs or silica coating MNPs were suspended in 10 mL of 8.6 M HCl and the mixture was stirred gently (100 rpm). After the desired time interval (0, 30 s, 1, 2, 4, 6, 8, 16, and 1440 min), 250 μL of supernatant was removed and added to 4.75 mL of distilled water. Then, absorbance of the supernatant at λ=333 nm was measured to quantified the dissolved iron.

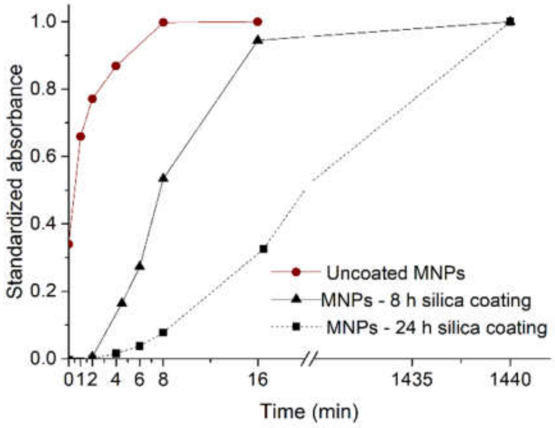

Figure S3. Acidic dissolution of bare MNPs and silica-coated MNPs.

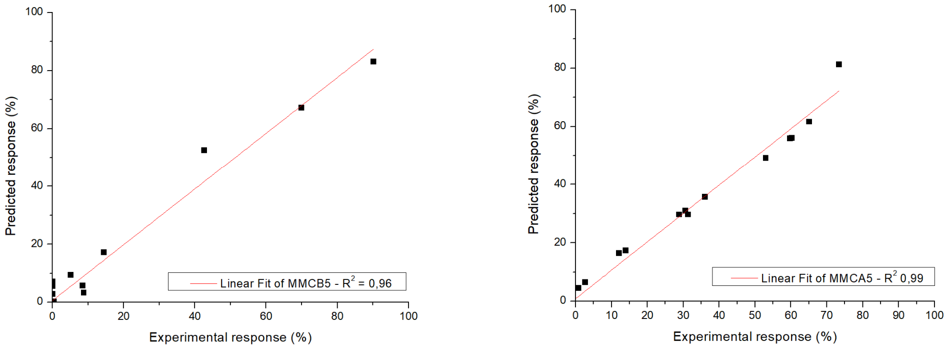

Figure S4. Correlation of experimental and predicted responses.

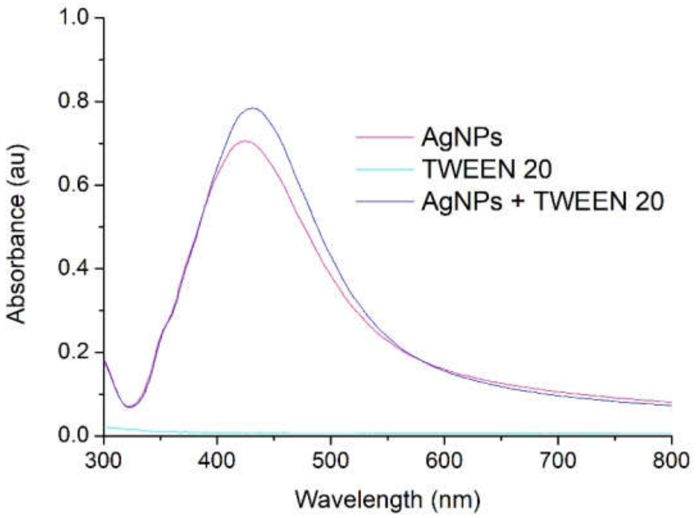

Figure S5. UV-Vis absorption spectra of AgNPs, tween 20, and AgNPs + tween 20.

Table S1. BBS chemical composition and functional groups [a].

Metal analysis						
**Si (%)**	**Fe (%)**	**Al (%)**	**Mg (%)**	**Ca (%)**	**K (%)**	**Na (%)**
12.14±0.07	1.03±0.02	0.59±0.01	1.67±0.25	4.86±0.61	1.18±0.07	0.06±0.01
**Microelements**						
**Cu (ppm)**	**Ni (ppm)**	**Zn (ppm)**	**Cr (ppm)**	**Pb (ppm)**	**Cd (ppm)**	**Hg (ppm)**
73±1	100±3	157±13	49±1	43±2	<0.02	<0.02
**General characteristics**				**Characteristics in aqueous solution 3 g L^{-1} [b]**		
**Moisture (%)**	**Ashes (%)**	**C (%)**	**N (%)**	**pH**	**Conducibility ($\mu S\ cm^{-1}$)**	**Surface tension γ**
3.69	31.2	39.94±0.35	4.82±0.14	9.02	429	56.8
**Concentration values as mole fraction of total C for functional groups and C types in BBS-GC by NMR analysis[c]**						
**Aliph**	**NR**	**COOH**	**OR**	**Ph**	**PhOH**	**Kt**
0.31	0.07	0.12	0.20	0.16	0.06	0.02

[a] From Nisticò, R. et al. From biowaste to magnet-responsive materials for water remediation from polycyclic aromatic hydrocarbons. Chemosphere 202, 686–693 (2018). [b] Maximum solubility is 100 g L^{-1} in water at ca. 60 °C. Solutions are stable in one week at different temperatures (tests conducted at 4 °C, 25 °C and 40 °C) with water hardness ≤ 40 °f. [c] Legends: Aliph = aliphatic groups, NR = amino groups, COOH = carboxylic acids, OR = alkoxy groups, Ph = aromatic phenyl groups, PhOH = phenoxy groups, Kt = ketones.

Table S2. Magnetic properties for MMCB5 and MMCA5.

Material	Coercitivity (G)	Remanence (emu g^{-1})	Saturation magnetization (emu g^{-1})
MMCB5	4.9	50	69.3
MMCA5	4	50	57.5

Table S3. Calculated weight loss after deposition of each polymer layer during the synthesis of MMCB5 and MMCA5 from TGA analysis.

Sample	Mass loss (%)	Sample	Mass loss (%)
MC	-	MC	-
MMCB2	0.04	MMCA2	-
MMCB3	0.2	MMCA3	0.4
MMCB4	1.7	MMCA4	2.6
MMCB5	3.5	MMCA5	2.4

Table S4. Zeta potential measurements of MMCB5 and MMCA5 at pH 5, 7, and 9.

	MMCA5			MMCB5		
pH	5	7	9	5	7	9
zeta potential (mV)	16	-22	-48	19	-26	-38

Table S5. Zeta potential measurements of AgNP1 and AgNP2 at pH 5, 7, and 9.

	AgNP1			AgNP2		
pH	5	7	9	5	7	9
zeta potential (mV)	-36	-41	-43	-18	-39	-39

Table S6. Control experiments. Results of magnetic iron oxide nanoparticles and chitosan in AgNPs removal.

Chitosan (mg L^{-1})	MNPs (mg L^{-1})	% RE*
10	10	2
5	5	12
5	10	12
2.5	5	11
20	50	7
50	50	1
50	100	3
50	366	6
50	1000	14

[AgNP1] = 13.5 mg L^{-1}, pH = 5. *RSD < 10%

Table S7. Analysis of variance (ANOVA) for %RE of MNFs.

Material	Sources	Sum of squares	Degree of freedom	Mean square	F_{value}	F_{table} (α=0.05%)	R^2	R^2_{adj}
MMCB5	Regression	10939.9	9	1215.55	42750.91	4.77	0.963	0.896
	Residues	420.99	5	84.2				
	Total	11360.9	14					
MMCA5	Regression	8174.37	9	908.26	461.45	4.77	0.971	0.919
	Residues	244.57	5	48.91				
	Total	8418.94	14					

Table S8. Analysis of variance (ANOVA) for the selected variables.

MMCB5			MMCA5		
Factor	Coefficient	P value	Factor	Coefficient	P value
b0	0.217	P≥0.05	b0	29.66	P≤0.001
b1	-1.109	P≤0.01	b1	-2.345	P≥0.05
b2	7.27	P≤0.001	b2	24.195	P≤0.001
b3	-41.325	P≤0.001	b3	-37.291	P≤0.001
b11	4.243	P≤0.001	b11	3.685	P≥0.05
b22	7.67	P≤0.001	b22	9.689	P≤0.05
b33	47.458	P≤0.001	b33	-0.396	P≥0.05
b12	10.219	P≤0.001	b12	-3.805	P≥0.05
b13	13.086	P≤0.001	b13	6.655	P≥0.05
b23	-22.753	P≤0.001	b23	-8.811	P≤0.05

Table S9. Zeta potential measurements of MNFs dispersion at pH 5 in the presence of different additives.

Material	[HA] (mg L^{-1})	[LAS] (mg L^{-1})	[TWEEN] (mg L^{-1})	Zeta potential (mV)
MMCA5	-	-	-	16
MMCA5	5	-	-	-17
MMCA5	-	10	-	4
MMCA5	-	-	10	13
MMCB5	-	-	-	12
MMCB5	5	-	-	-20
MMCB5	-	10	-	-2
MMCB5	-	-	10	5
